# Anmyungambi Decoction Ameliorates Obesity through Activation of Non-Shivering Thermogenesis in Brown and White Adipose Tissues

**DOI:** 10.3390/antiox12010049

**Published:** 2022-12-26

**Authors:** Woo Yong Park, Gahee Song, Mina Boo, Hyo In Kim, Ja Yeon Park, Se Jin Jung, Minji Choi, Beomsu Kim, Young Doo Kim, Myung-Ho Kim, Kwan-Il Kim, Hyun Jeong Kwak, Jungtae Leem, Jae-Young Um, Jinbong Park

**Affiliations:** 1 Department of Pharmacology, College of Korean Medicine, Kyung Hee University, Seoul 02447, Republic of Korea; 2 Department of Science in Korean Medicine, Graduate School, Kyung Hee University, Seoul 02447, Republic of Korea; 3 Department of Surgery, Beth Israel Deaconess Medical Center, Harvard Medical School, Boston, MA 02215, USA; 4 Jaonmi Korean Medicine Clinic, Seoul 06734, Republic of Korea; 5 Liver Center, Gastrointestinal Division, Massachusetts General Hospital, Harvard Medical School, Boston, MA 02215, USA; 6 Division of Allergy and Immune Division of Allergy, Immune and Respiratory System, Department of Internal Medicine, College of Korean Medicine, Kyung Hee University, Seoul 02447, Republic of Korea; 7 Department of Life Science, College of Natural Sciences, Kyonggi University, Suwon 16227, Republic of Korea; 8 College of Korean Medicine, Wonkwang University, Iksan 54538, Republic of Korea

**Keywords:** Anmyungambi decoction, obesity, brown adipose tissue, beige adipocytes, non-shivering thermogenesis, lipolysis, AMP-activated protein kinase, reactive oxygen species

## Abstract

Obesity is a burden to global health. Non-shivering thermogenesis of brown adipose tissue (BAT) and white adipose tissue (WAT) is a novel strategy for obesity treatment. Anmyungambi (AMGB) decoction is a multi-herb decoction with clinical anti-obesity effects. Here, we show the effects of AMGB decoction using high-fat diet (HFD)-fed C57BL6/J mice. All four versions of AMGB decoction (100 mg/kg/day, oral gavage for 28 days) suppressed body weight gain and obesity-related blood parameters in the HFD-fed obese mice. They also inhibited adipogenesis and induced lipolysis in inguinal WAT (iWAT). Especially, the AMGB-4 with 2:1:3:3 composition was the most effective; thus, further studies were performed with the AMGB-4 decoction. The AMGB-4 decoction displayed a dose-dependent body weight gain suppression. Serum triglyceride, total cholesterol, and blood glucose decreased as well. In epididymal WAT, iWAT, and BAT, the AMGB-4 decoction increased lipolysis markers. Additionally, the AMGB-4 decoction-fed mice showed an increased non-shivering thermogenic program in BAT and iWAT. Excessive reactive oxygen species (ROS) and suppressed antioxidative factors induced by the HFD feeding were also altered to normal levels by the AMGB-4 decoction treatment. Overall, our study supports the clinical use of AMGB decoction for obesity treatment by studying its mechanisms. AMGB decoction alleviates obesity through the activation of the lipolysis–thermogenesis program and the elimination of pathological ROS in thermogenic adipose tissues.

## 1. Introduction

Obesity is a major health threat worldwide. Defined by an accumulation of excess energy in the form of lipid, obesity is a risk factor of various fatal diseases, including type 2 diabetes [1], cardiovascular diseases [2], and even cancer [3]. Body mass index (BMI) is used as a simple index: for adults, the World Health Organization (WHO) defines overweight as BMI ≥ 25 and obesity as BMI ≥ 30. In 2016, around 13% of adults all over the world were obese. More seriously though, overweight and obesity among children and adolescents have increased dramatically [4]. Since the incidence of obesity is rapidly increasing, especially in developed countries, obesity management is gaining great interest from clinicians and researchers.

The imbalance between energy intake and expenditure leads to an accumulation of lipid, in particular among white adipose tissue (WAT). On the other hand, the contribution of brown adipose tissue (BAT) to energy expenditure has been widely accepted since its unexpected rediscovery in 2007 [5]. BAT was first characterized in 1960s, but it has been understood for decades that it only exists in newborn babies but not adults [6]. While WAT stores excess energy as triglyceride (TG), BAT induces uncoupled mitochondrial respiration via the activation of uncoupling protein 1 (UCP1) [7]. This special type of mitochondrial action requires fatty acid as fuel and results in heat [8]. Moreover, later studies have discovered an inducible, recruitable type of brown-like adipocytes within WAT, the so-called beige adipocytes [9]. In thermogenic adipose tissues, free fatty acids (FFAs) fuel the UCP1-dependent non-shivering thermogenesis [10]. Thus, lipid-consumed thermogenesis is considered a potentially efficient mechanism for energy dissipation. Importantly, adipocytes have an overlapping but distinct gene expression patterns [11]. At present, the thermogenic capacity of brown and beige adipocytes attracts particular interests, considering the world-wide burden of obesity.

An attractive feature of these thermogenic adipocytes is that they respond to pharmacological stimuli. Several pharmacological agents are shown to activate BAT thermogenesis [12,13] and recruit beige adipocytes in inguinal WAT (iWAT) [14,15]. Dietary and nutritional support can also have similar effects [16]. In such aspects, natural products and herbal medicine have the potential to induce and, thus, treat metabolic diseases through non-shivering thermogenesis-related mechanisms. Several plant extracts and their compounds (e.g., flavonoids, catechins, and alkaloids) have been shown to have positive effects on non-shivering thermogenesis [17].

Anmyungambi (AMGB) decoction is an herbal decoction composed of four herbs, which is prescribed to treat obesity in traditional Korean medicine. Glycine Semen Preparata (SP), Gardeniae Fructus (GF), Phellodendri Cortex (PC), and Ephedrae Herba (EH) are the four herbal constituents of AMGB decoction, and different proportions are used based on the patient’s body status. AMGB decoction was developed to reduce the adverse effects of EH based on the clinical experience of a Korean physician specialized in herbal medicine. EH was intended to be the major herb with anti-obesity effects, while SP and PC were added to reduce the side effects of EH. PC was added to manage the appetite of patients. Our previous study has discussed the clinical outcomes of AMGB decoction in 27 obese individuals [18]. In addition, through network pharmacological approaches, we have defined several target mechanisms of AMGB decoction. Our previous work anticipates ABGM to not only control the adverse effects of EH but also regulate obesity-associated mechanisms, including insulin resistance, lipid metabolism, and hepatocyte injury [19]. Based on our previous reports, we determine in this study the in vivo effect and related mechanisms of AMGB decoction, focusing on its regulatory effect on non-shivering thermogenesis in particular, using a high-fat diet (HFD)-fed obese mouse model.

## 2. Materials and Methods

### 2.1. Preparation of AMGB Decoctions

As reported in the previous case series report, the most frequently used composition ratios of herbs comprising AMGB decoction was utilized in the current experimental study [18]. Since AMGB decoction is prescribed in several proportions according to the individual’s body condition, we selected 4 different versions of the decoction. AMGB-1: SP 4 g, GF 2 g, PC 1 g, and EH 3 g (total 10 g per preparation); AMGB-2: SP 4 g, GF 2 g, PC 3 g, and EH 3 g (total 12 g per preparation); AMGB-3: SP 6 g, GF 3 g, PC 3 g, and EH 6 g (total 18 g per preparation); and AMGB-4: SP 4 g, GF 2 g, PC 6 g, and EH 6 g (total 18 g per preparation). A1 at 150 g, A2 at 180 g, A3 at 270 g, or A4 at 270 g was boiled in 6.8 L of water at 110 °C for 150 min. Then, the boiled concentrates were freeze-dried after filtration by 0.5 μm-pore size membrane filters. The remaining powder was collected and weighed; the yield of AMBG-1, AMBG-2, AMBG-3, and AMBG-4 was 7.33%, 6.67%, 5.56%, and 5.93%, respectively. The validation of GF (geniposide content 2.3%), PC (berberine content 1.1%) and EH (ephedrine and pseudoephedrine content 1.7%) was performed by the National Institute for Korean Medicine Development (Gyeongsan, Republic of Korea).

### 2.2. Animal Study

All animal procedures were performed according to a protocol approved by the Animal Care and Use Committee of the Institutional Review Board of Kyung Hee University (confirmation number: KHUASP (SE)-19-405). In brief, 7-week-old male C57BL/6J mice were purchased from Daehan Biolink Co., Eumsung, Republic of Korea, and were kept for 1 week for acclimatization prior to further studies. The mice were fed a HFD for additional 6 weeks to induce obesity. Normal chow diet-fed lean mice (*n* = 7~8) were used as a normal control group (ND group). Then, for the proportional study, HFD-fed mice were divided into 5 groups (n = 7 per group). The control mice were fed with PBS and the experimental groups were fed with 200 mg/kg of AMGB-1, AMGB-2, AMGB-3, or AMGB-4 daily for 4 weeks. For the dose study of AMGB-4, HFD-fed mice were divided into 4 groups (n = 8 per group). The control mice were fed with PBS and the experimental groups were fed with 50, 100, or 200 mg/kg of A4 daily for 4 weeks. The mice were maintained on a 12 h light/12 h dark cycle at 24 °C in a specific pathogen-free environment during the whole period. After the experiment was over, the mice were sacrificed by cervical dislocation under CO_2_ asphyxiation, and the liver, epididymal white adipose tissue (eWAT), iWAT, and BAT were harvested and weighed. The tissues were either snap frozen and stored at −80 °C for further analysis or fixed in formalin for histological studies. The ND and HFD compositions are described in Table 1.

### 2.3. Serum Analysis

Serum TG, glucose, aspartate aminotransferase (AST), alanine transaminase (ALT), total cholesterol, high-density lipoprotein (HDL) cholesterol, low-density lipoprotein (LDL) cholesterol, and blood urea nitrogen (BUN) levels were measured at the Seoul Medical Science Institute (Seoul Clinical Laboratories, Seoul, Republic of Korea). In brief, AST and ALT were analyzed using the enzymatic colorimetric absorbance assays according to the International Federation of Clinical Chemistry. Glucose was measured using the UV assay based on the hexokinase enzymatic method. Creatinine was measured based on the Jaffé method. BUN was measured using the kinetic test with urease and glutamate dehydrogenase. Cholesterol levels were measured using the homogenous enzymatic colorimetric method. All assays were performed using a Cobas c 502 system with an ISE module, Serial.no: 18J4-06, 18Y2-06 (Roche Diagnostics, Indianapolis, IN, USA).

### 2.4. Hematoxylin and Eosin (H&E) Staining

H&E staining was performed as described previously [20]. Briefly, the tissues were fixed in 10% formalin and embedded in paraffin, and then cut into 5 μm sections. The tissue sections were placed on a glass slide and stained with H&E. Microscopic examinations were performed and photographs were taken under the EVOSR Cell Imaging System (Thermo Scientific, Waltham, MA, USA).

### 2.5. Western Blot Assay

Protein extraction and Western blot analysis were performed as previously reported [20]. Briefly, the protein extracts from the homogenized tissues were lysed in a radioimmunoprecipitation assay (RIPA) buffer (Cell Signaling Technology, Danvers, MA, USA), and the lysates were resolved by sodium dodecyl sulfate–polyacrylamide gel electrophoresis and transferred onto polyvinylidene difluoride membranes. Then, the membranes were incubated with primary antibodies (1:1000), followed by incubation with horseradish peroxidase-conjugated secondary antibodies (1:10,000). Protein signals were obtained using the ECL advance kit (GE Healthcare Life Sciences, Seoul, Republic of Korea), and chemiluminescence intensity was analyzed using the ImageJ software (National Institutes of Health, Bethesda, MD, USA). The antibodies used in this study are described in Table 2.

### 2.6. Blood Glucose Measurement

The mice were fasted overnight, and blood glucose was measured from the blood obtained by removing the distal 2 mm of the tail. A glucometer Accu-Chek Performa (Roche Diagnostics, Mannheim, Germany) was used as previously described [21].

### 2.7. FFA Level

An EZ-Free Fatty Acid Assay Kit (Cat. No. DG-FFA100) was used to measure the FFA levels in BAT and iWAT according to the manufacturer’s instructions (DoGenBio Co., Ltd., Seoul, Republic of Korea).

#### Catalase Activity

An EZ-Catalase Assay Kit (Cat. No. DG-CAT400) was used to measure the catalase activity in BAT and iWAT according to the manufacturer’s instructions (DoGenBio Co., Ltd.).

### 2.8. Statistical Analysis

All data are expressed as the mean ± standard error of the mean (SEM) of 3 or more independent experiments. Statistical differences were calculated by one-way ANOVA using Prism 8 (GraphPad Software, San Diego, CA, USA). *p*-values of < 0.05 were considered statistically significant.

## 3. Results

### 3.1. Various Versions of AMGB Decoction Inhibit Body Weight Gain and Obesity-Related Serum Parameters in HFD-Induced Obese Mice

**Figure 1:**
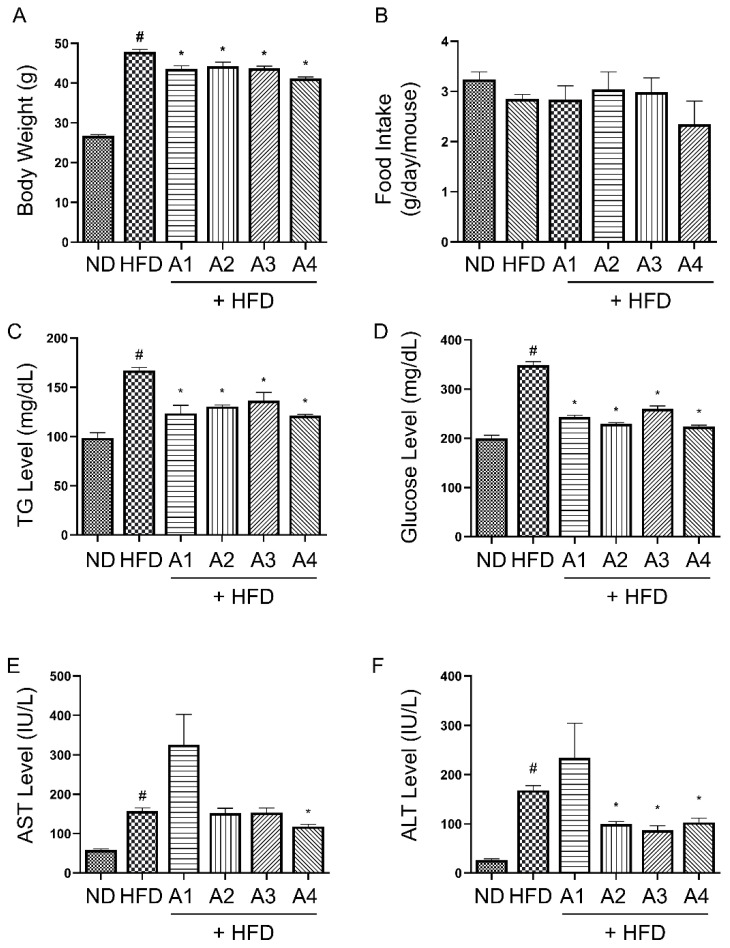
Effect of different versions of AMGB decoction on obesity-related parameters in HFD-induced obese C57BL6/J mice. (**A**) Body weight at sacrifice; (**B**) average food intake; serum levels of (**C**) TG, (**D**) glucose, (**E**) AST, and (**F**) ALT. # *p* < 0.05 vs. ND-fed control mice; * *p* < 0.05 vs. HFD-fed control mice.

After the induction of obesity by HFD feeding for six weeks, the mice showed an average body weight of 38.85 ± 2.24 g (vs. 25.79 ± 1.34 g in ND-fed mice). Through the oral administration of four different proportional versions of AMGB decoction, the mice showed decreased body weight. The average body weight of each group at the moment of sacrifice is as follows: ND, 26.74 ± 0.32 g; HFD, 48.88 ± 0.66 g; AMGB-1, 44.63 ± 0.75 g; AMGB-2, 45.25 ± 1.05 g; AMGB-3, 44.75 ± 0.52 g; and AMGB-4, 43.13 ± 0.43 g (Figure 1A). Average food intake shows a slight tendency of decreasing without statistical difference (Figure 1B). The AMGB decoctions all significantly suppress the increase in TG and glucose (Figure 1C,D). In addition, hepatotoxicity markers, such as AST and ALT, are decreased by the A4 decoction, indicating an alleviation of liver steatosis. The AMGB-1 fails to decrease both AST and ALT, while the AMGB-2 and the AMGB-3 only suppress ALT levels (Figure 1E,F).

### 3.2. Various Versions of AMGB Decoction Suppress Adipogenesis and Increase Lipolysis in HFD-Induced Obese Mice

**Figure 2:**
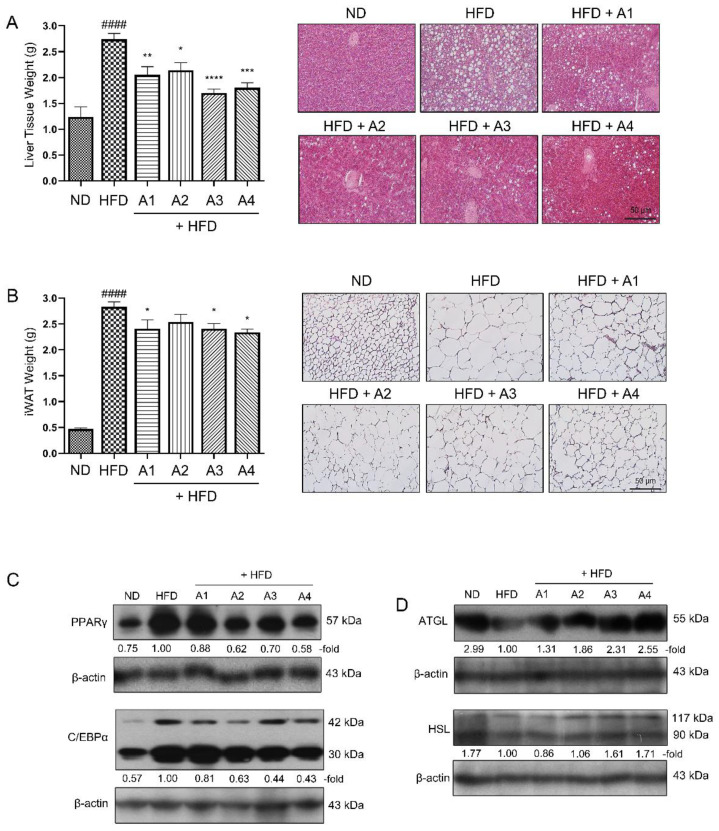
Effect of different versions of AMGB decoction on liver and adipose tissue of HFD-induced obese C57BL6/J mice. (**A**) Liver tissue weight was measured and histological analysis was performed using H&E staining. (**B**) iWAT weight was measured and histological analysis was performed using H&E staining. Western blot assays were performed in the iWAT of the mice to verify changes in (**C**) adipogenesis markers and (**D**) lipolysis markers. #### *p* < 0.05 vs. ND-fed control mice; * *p* < 0.05, ** *p* < 0.01, *** *p* < 0.001, **** *p* < 0.0001 vs. HFD-fed control mice.

The AMGB decoctions decreases the weight and lipid accumulation of the liver tissues (Figure 2A). Similar effects were observed in the adipose tissue as well. iWAT is the subcutaneous fat depot which accumulates excessive calorie in the form of TG [22]. The HFD administration led to increased lipid accumulation in iWAT. However, the 4-week feeding of the AMGB decoctions notably decreased the lipid droplet sizes (Figure 2B). Such effect was due to suppressed adipogenesis and increased lipolysis. As shown in Figure 2C, all four versions of AMGB decoctions inhibit PPARγ and C/EBPɑ, the two key regulators of adipogenesis. In addition, the AMGB decoctions induce the levels of ATGL and HSL (Figure 2D). Based on these findings, we could conclude that the most frequently prescribed proportions of AMGB decoction had significant anti-obese effects through the suppression of adipogenesis and the induction of lipolysis. The overall effect was greatest for the AMGB-4; thus, further studies were continued with this version of AMGB decoction.

### 3.3. AMGB-4 Decoction Alleviates HFD-Induced Obesity and Hepatic Steatosis in Mice

**Figure 3:**
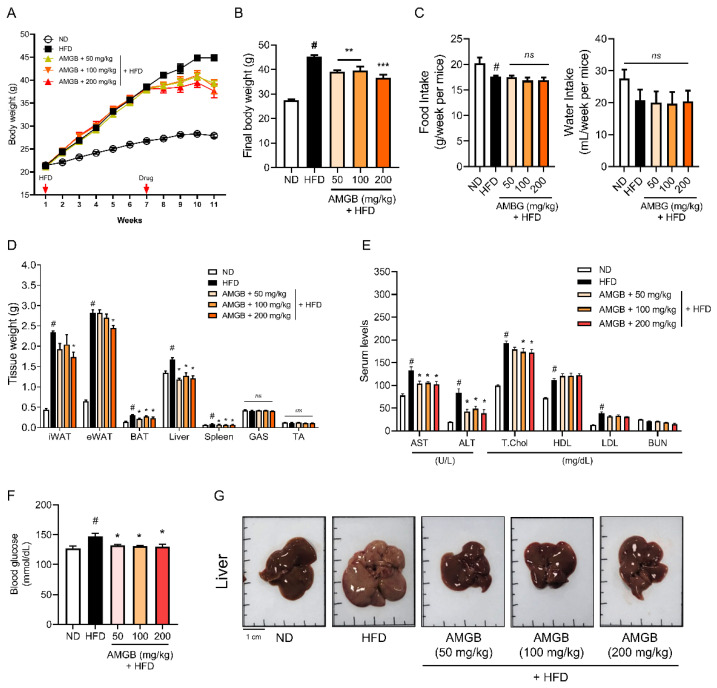
Effect of the AMGB-4 decoction on obesity-related parameters in HFD-induced obese C57BL6/J mice. (**A**) The weekly body weight change and (**B**) final body weight at sacrifice were measured. (**C**) Average food intake and water consumption were measured. (**D**) Tissue weight was measured. (**E**) Serum analysis on AST, ALT, total cholesterol, HDL cholesterol, LDL cholesterol, and BUN was performed. (**F**) Blood glucose level was measured. (**G**) Representative pictures of the liver tissues of each group are presented. # *p* < 0.05 vs. ND-fed control mice; * *p* < 0.05, ** *p* < 0.01, *** *p* < 0.001 vs. HFD-fed control mice. ns: not significant.

To investigate the dose-dependent effect of the AMGB-4 decoction, we conducted another animal study with three different doses of AMGB-4 administration. The AMGB-4 decoction shows a dose-dependent inhibition of body weight gain (Figure 3A,B). Food or water intake is unchanged (Figure 3C). As shown in Figure 3D, iWAT and eWAT weights are decreased by the AMGB-4 treatment only at the highest dose (200 mg/kg/day). Liver, BAT, and spleen weights are suppressed at all doses of the AMGB-4, and muscle weights (gastrocnemius and tibialis anterior) are unaffected in all groups. Serum analysis (Figure 3E) shows that the AMGB-4 administration decreases liver steatosis. The AMGB-4 at a dose of 100 and 200 mg/kg/day significantly suppresses total cholesterol increase. Increase in HDL or decrease in LDL cholesterols is slight but insignificant. BUN is unchanged in all groups. Blood glucose decreases at all doses (Figure 3F). The AMGB-4 decoction inhibits lipid accumulation in the liver, as determined by the color and size (Figure 3G).

### 3.4. AMGB-4 Decoction Induces Lipolysis in the eWAT of HFD-Induced Obese Mice

**Figure 4:**
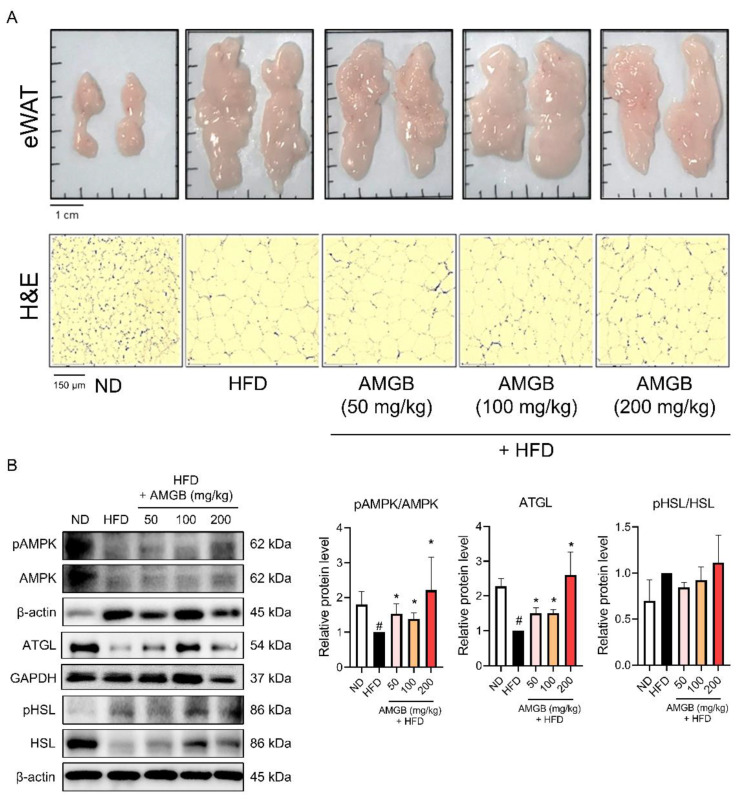
Effect of the AMGB-4 decoction on the eWAT of HFD-induced obese C57BL6/J mice. (**A**) Representative pictures of the eWAT otological analysis was performed using H&E staining. (**B**) Western blot assays were performed in the eWAT of the mice to verify changes in lipolysis markers. # *p* < 0.05 vs. ND-fed control mice; * *p* < 0.05 vs. HFD-fed control mice.

Visceral WAT, including eWAT, is the largest fat depot [23,24]. We studied whether the AMGB-4 could induce changes in the eWAT of the mice. Macroscopic observations and histological analysis suggest that the AMGB-4 decoction suppresses excessive lipid accumulation in eWAT (Figure 4A). Amount of lipid is regulated through the balance of synthesis and expenditure of lipid, also known as adipogenesis (or lipogenesis) and lipolysis [25]. We investigated the changes in lipolysis-related factors, such as AMPKα [26], ATGL, and HSL [27], in the eWAT of the mice (Figure 4B). The AMGB-4 treatment significantly increases the phosphorylation of AMPKα and ATGL. The phosphorylation of HSL shows a tendency to increase by the AMGB-4, however, the statistical analysis determines the changes as insignificant.

### 3.5. AMGB-4 Decoction Induces Non-Shivering Thermogenesis in the BAT of HFD-Induced Obese Mice

**Figure 5:**
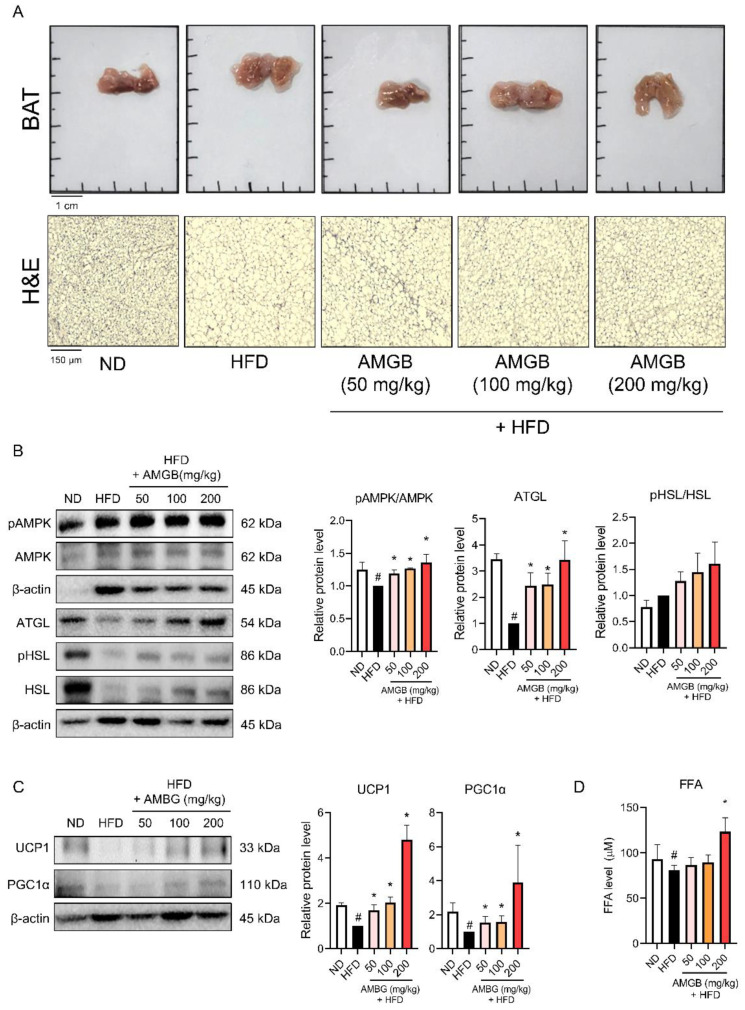
Effect of the AMGB-4 decoction on the BAT of HFD-induced obese C57BL6/J mice. (**A**) Representative pictures of the BAT of each group are presented, and histological analysis was performed using H&E staining. Western blot assays were performed in the BAT of the mice to verify changes in (**B**) lipolysis markers and (**C**) non-shivering thermogenesis markers. (**D**) The FFA level in the tissue lysate was measured. # *p* < 0.05 vs. ND-fed control mice; * *p* < 0.05 vs. HFD-fed control mice.

BAT is the main organ of non-shivering thermogenesis [6]. This animal study revealed that the AMGB-4 decoction altered non-shivering thermogenesis in the BAT. The HFD-induced lipid accumulation in BAT decreases upon the AMGB-4 treatment (Figure 5A). Moreover, phosphorylation of lipolysis-related factors, including AMPKα, ATGL, and HSL (n.s.), is induced in the BAT of the AMGB-4-treated mice (Figure 5B), indicating an activation of energy expenditure and lipolysis. Consistently, UCP1 and PGC1α, the controllers of non-shivering thermogenesis, are both increased by the AMGB-4 treatment (Figure 5C). Further lipolytic activities induced by the AMGB-4 were determined by measuring the FFA level in the tissue. As shown in Figure 5D, AMGB at the highest dose of 200 mg/kg/day revokes the HFD-induced decrease in BAT FFA, suggesting its lipolytic activity.

### 3.6. AMGB-4 Decoction Induces Non-Shivering Thermogenesis in the iWAT of HFD-Induced Obese Mice

**Figure 6:**
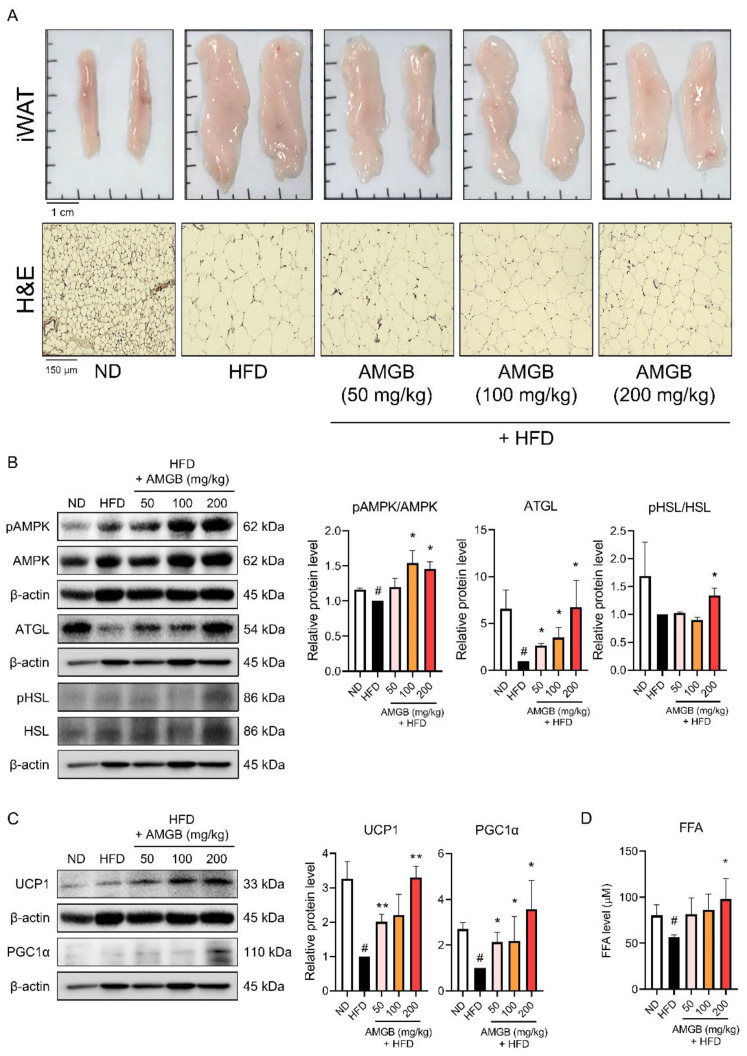
Effect of the AMGB-4 decoction on the iWAT of HFD-induced obese C57BL6/J mice. (**A**) Representative pictures of the iWAT of each group are presented, and histological analysis was performed using H&E staining. Western blot assays were performed in the iWAT of the mice to verify changes in (**B**) lipolysis markers and (**C**) non-shivering thermogenesis markers. (**D**) The FFA level in the tissue lysate was measured. # *p* < 0.05 vs. ND-fed control mice; * *p* < 0.05, ** *p* < 0.01 vs. HFD-fed control mice.

Studies have reported that cold stimuli and pharmacological/nutritional interventions can induce browning of white adipocytes, and this mainly takes place in iWAT [14,15]. iWAT is also a fat storage depot in obesity [22]. Thus, we investigated the changes in iWAT induced by the AMGB-4 treatment. The results show that the AMGB-4 decoction can highly suppress lipid accumulation in iWAT (Figure 6A). Similar to the effects in BAT, the phosphorylation levels of AMPK and ATGL are also increased by the AMGB-4 in the iWAT (Figure 6B). Additionally, UCP1 and PGC1α are significantly increased in the iWAT of the AMGB-4-fed mice, showing that the AMGB-4 can induce browning and non-shivering thermogenesis of white adipocytes (Figure 6C). Similar to the results shown in BAT, 200 mg/kg/day of the AMGB-4 treatment significantly increases the FFA level in iWAT (Figure 6D). These findings suggest that the AMGB-4 induces browning in iWAT and increases lipolysis and non-shivering thermogenesis.

### 3.7. AMGB-4 Decoction Reduces Pathologic Oxidative Stress by Increasing The Nuclear Factor Erythroid 2–Related Factor 2 (NRF2)-Heme Oxygenase-1 (HO-1) Axis in iWAT and BAT of HFD-Induced Obese Mice

**Figure 7:**
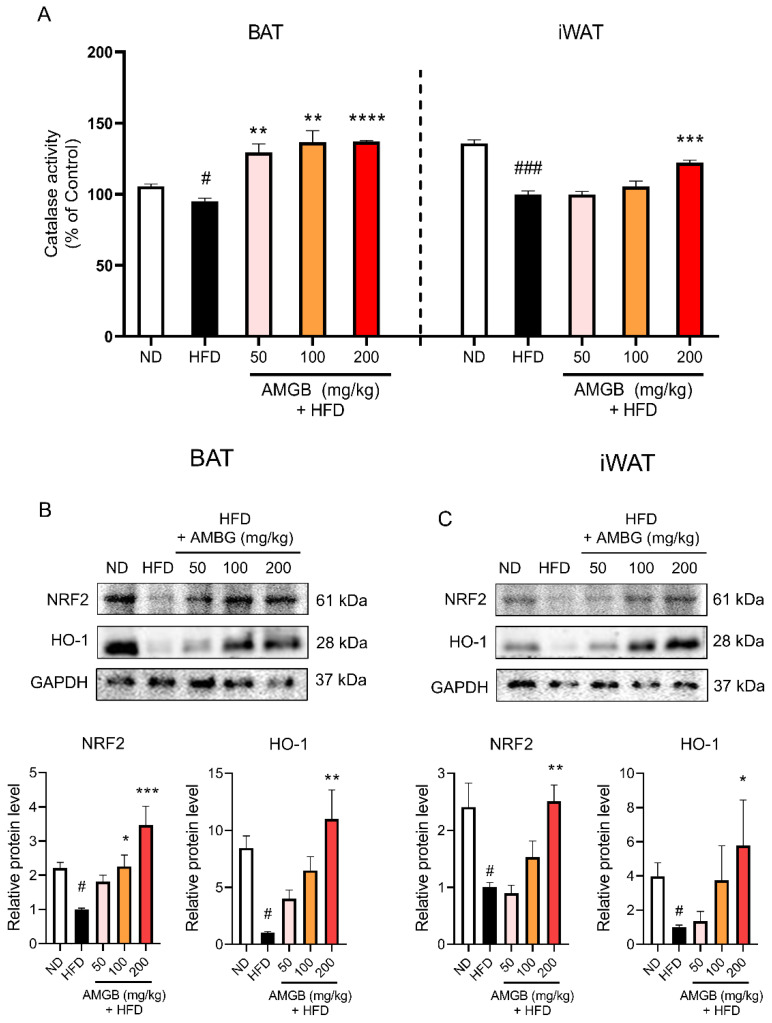
Antioxidative effect of the AMGB-4 decoction in the BAT and iWAT of HFD-induced obese C57BL6/J mice. (**A**) Oxidative stress was determined by measuring catalase activity in BAT and iWAT. Western blot assays were performed in the (**B**) BAT and (**C**) iWAT of the mice to verify changes in antioxidative markers. # *p* < 0.05, ### *p* < 0.001 vs. ND-fed control mice; * *p* < 0.05, ** *p* < 0.01, *** *p* < 0.001, **** *p* < 0.0001 vs. HFD-fed control mice.

Non-shivering thermogenesis is closely related to reactive oxygen species (ROS) and related oxidative stress within the adipose tissue [28]. Thus, we measured the catalase activities [29] in these tissues to see whether oxidative stress is related to the effect of the AMGB-4. As shown here, we observed that all four does of the AMGB-4 treatment increase the catalase activity in BAT and the dose of 200 mg/kg/day increases the catalase activity in iWAT (Figure 7A), implying the AMGB-4 reduces pathological oxidative stress within both tissues. We measured the protein levels of the antioxidant factors, NRF2 and HO-1 [30], to determine the antioxidant properties of the AMGB-4. While HFD notably decreases the levels of NRF2 and HO-1, the AMGB-4 treatment revokes such changes (Figure 7B). As observed in the BAT, similar antioxidative effects by the AMGB-4 are shown in the iWAT. NRF2/HO-1 expression also increases (Figure 7C).

## 4. Discussion

AMGB decoction is composed of four herbs: SP (fermented beans of *Glycine max*), GF (dried fruit of *Gardenia jasminoides*), PC (dried bark of *Phellodendron amurense*), and EH (dried above-ground part of *Ephedra sinica*). EH is one of the most frequently prescribed herbal medicine to treat obesity [31]; however, a large number of patients complain of insomnia or psychological anxiety as side effects [32]. As Zhizichi decoction, which is consisted of SP and GF, is widely used for insomnia treatment in clinical practice [33], the two herbs and the obesity-controlling herb EH were combined. Then, PC was added to control appetite to enhance the anti-obesity effect [34]. Although AMGB decoction has been prescribed to treat obesity in local Korean medical clinics, only limited evidence is available besides clinical data. Thus, our recent effort aimed to elucidate the underlying mechanisms of its anti-obesity effect. By reviewing case series [18] and including AMGB decoction in a systemic review/meta-analysis [35], its clinical effect was validated. In addition, through network pharmacological approaches [19], we were able to determine the target of interests. Furthermore, several attempts may also provide scientific evidence for the beneficial effect of AMGB decoction, particularly by the following reports on its composing herbs.

SP, also known as soybeans, is frequently consumed as a sauce in Korea’s traditional cuisines in the form of a paste (Doenjang or Chungkookjang). The anti-obesity effect of SP is well described in the literature. Several studies reported the anti-obesity effect of SP in vivo [36,37,38,39,40,41]. Cha et al. also showed that SP is clinically effective in a 12-week randomized clinical trial [42]. GF, the fruit of *Gardenia jasminoides*, has been consumed as a herbal tea or used as a yellow dye for ages [43]. Moreover, in traditional Korean medicine, GF is considered a herbal medicine which can alleviate inflammation. Our previous study demonstrated the anti-obesity effect of GF by inducing mitochondrial activation and non-shivering thermogenesis [20]. Shin and Huh reported that GF consumption combined with exercise can improve obesity in middle-aged obese women by affecting hormones that regulate energy metabolism [44]. Its constituents, geniposide [45,46] and genipin [47,48,49], are also known to display anti-obesity effects in vivo and in vitro. Although there is no report available in PubMed on the anti-obesity effect of PC, evidence suggests the possibility. Wang et al. demonstrated that evodiamine, an active compound of PC, improves obesity independently to UCP1 using knockout mice [50]. Evodiamine also controls adipocyte differentiation [51,52] and improves glucose metabolism in mice [53]. Berberine is also a constituent of PC. Berberine is well known to induce activation of brown adipocytes and browning of white adipocytes [54,55]. EH and caffeine supplement showed anti-obesity effects in a 6-month randomized safety and efficacy trial [56]. A meta-analysis of clinical trials suggested its effectiveness in short-term weight loss [57]. The anti-obesity effect of EH have also been demonstrated in lab studies. Song et al. [58] and Lee et al. [59] reported that EH reduced obesity and hyperglycemia in HFD-fed mice, and Wang et al. suggested that gut microbiota was related to such effect [60]. Recently, EH was shown to induce browning in murine and human white adipocytes [61]. Overall, these clues suggest the underlying mechanisms of the anti-obesity effect of AMGB decoction.

Adipogenesis is the main pathway of lipid accumulation. Excessive, leftover energy is transformed into the form of lipid through a process called adipogenesis/lipogenesis and then stored in WAT and liver. Therefore, adipogenesis and related pathways have long been the therapeutic targets for obesity control [62]. PPARγ is a nuclear receptor highly expressed in adipocytes, which is known as one of the key factors of adipogenesis [63]. PPARγ is essential for adipogenesis. Knockout of PPARγ results in the loss of mature adipocytes and adipose tissue formation [64]. Consistently, no factor has been shown to drive adipogenesis without the cooperation of PPARγ [65]. Although not as important as PPARγ, C/EBPα is also considered a key regulator of adipogenesis [66]. C/EBPα and PPARγ both participate in the adipogenesis process, with PPARγ acting as the main driver [67], while C/EBPα binds to the PPARγ promoter and induces its expression [68]. It is well established that PPARγ and C/EBPα cooperate through mutual inductions [69,70]. In this study, AMGB decoctions, in various proportions, effectively suppress these two regulators of adipogenesis in iWAT (Figure 2C). Such results suggest that AMGB decoction plays an inhibitory role during lipid accumulation at the adipocyte differentiation level.

Non-shivering thermogenesis is a promising target strategy for obesity treatment gaining recent interest. The thermogenic potential of activated BAT can burn 20 kcal a day in an individual, which is 15% more than ones without activation [71]. White adipocytes are characterized by large intracellular lipid droplets and few mitochondria, with excessive energy stored in the form of TG. On the other hand, BAT consists of brown adipocytes, which are morphologically distinct from white adipocytes and contain abundant mitochondria and multilocular lipid droplets. These brown adipocytes work as the main organ of non-shivering thermogenesis in mammals [72]. Originally, it was accepted as a fact that adult humans do not have active BAT. However, starting from 2007, studies reported that adults also possess functional BAT [5,73,74,75]. It was further verified that BAT activity is negatively correlated with BMI and age, and positively correlated with glucose tolerance and insulin sensitivity [76]. Strikingly, brown-like adipocytes were later described [9,77]. These were named “brite” or “beige” adipocytes. Although derived from a different origin, beige adipocytes share several characteristics with classical brown adipocytes, such as abundant mitochondria and excessive energy expenditure upon cold stimuli [78]. Beige-induced iWAT are both characterized by multilocular morphology of lipid droplets, and our data show that different versions of AMGB decoctions can reduce the weight of iWAT and also the size of lipid droplets within it (Figure 2B).

UCP1 is the main factor of non-shivering thermogenesis which localizes at the inner membrane of mitochondria and is abundantly expressed in brown adipocytes. The importance of UCP in non-shivering thermogenesis has been described precisely. Genetic deletion of UCP1 leads to disrupted uncoupling of mitochondrial proton; thus, when exposed to cold, UCP1-knockout mice show impaired thermogenesis [79] and defective adaptive adrenergic signaling [80]. Interestingly though, UCP-knockout mice do not display obese phenotypes unless they are kept under a thermoneutral environment around 30 °C [81]. Yet, evidence clearly shows that UCP1 is essential for non-shivering thermogenesis of mitochondria. In this study, AMGB decoction increases the UCP1 levels in both BAT (Figure 5C) and iWAT (Figure 6C), indicating the activation of BAT thermogenesis and also the recruitment/activation of beige adipocytes within the iWAT. Similarly, PGC1α levels are increased in both adipose tissues with thermogenic potential (Figure 5C and Figure 6C). While UCP1 acts as the main protein of uncoupling respiration and subsequent non-shivering thermogenesis, PGC1α is a transcription factor which centers in the network of mitochondrial biogenesis and brown/beige adipocyte differentiation [82].

While non-shivering thermogenesis is a complicated process involving several pathways, one critical factor is the fuel supply through lipolysis. Lipolysis describes TG hydrolysis. The importance of lipolysis was proven by Whitehead, showing that TG hydrolysis is essential for cellular uptake of fatty acids [83]. Upon cold stimulation, β-adrenergic signaling is activated in BAT. When such an increase of lipolysis takes place, the generated free fatty acids activates UCP1-mediated non-shivering thermogenesis [84]. Similarly, cold-induced browning of iWAT accompanies the increased oxidation of fatty acids through UCP1-mediated non-shivering thermogenesis [85]. Triacylglycerol (TAG) is catalyzed by the lipases ATGL and HSL. These two steps are recognized as the regulatory steps of lipolysis [27], since in adipose tissues, ATGL and HSL are responsible for more than 90% of TAG hydrolysis [86]. We see a significant increase in ATGL expression and HSL phosphorylation by the AMGB decoction treatment, in the eWAT (Figure 4B), BAT (Figure 5B), and iWAT (Figure 6B) of the HFD-fed obese mice. Such results directly support the beneficial effect of AMGB decoction in decreasing lipid accumulation in both white adipose tissues, and also indirectly indicate the activation of non-shivering thermogenesis in brown and beige adipocytes. BAT activation stimulates TG clearance by driving lipolysis [87]. Interestingly, lipolysis in BAT itself is not always required, but the remote lipolysis in WAT is rather more important [88].

AMPK is an energy sensor and key regulator of metabolic homeostasis. AMPK is a heterotrimeric protein complex consisting of α, β, and γ subunits. In adipose tissue, the α1 catalytic subunit of AMPK is the predominant isoform and is responsible for the functional AMPK activity [89]. AMPK stimulates pathways involved in energy expenditure and suppresses pathways involved in energy storage; in other words, it stimulates catabolism and suppresses anabolism. BAT and iWAT thermogenesis are closely linked to AMPK. Intracellular action of AMPK in brown adipocytes takes place when local energy deficit exists. In addition, hypothalamic AMPK regulates the whole-body metabolism and can control the sympathetic nerve system to secrete norepinephrine, which, in turn, activates BAT thermogenesis [90]. AMPK also plays a vital role in WAT browning in response to cold stimuli or pharmacological agents [91]. Additionally, AMPK is known to participate in lipase activities, in both ATGL and HSL, and to involve in the overall lipolysis process in the adipose tissue [26]. Here, we show that AMPK activity is increased by AMGB decoction, in BAT and both WATs (Figure 4B, Figure 5B and Figure 6B). These data fall in line with the inducible effect of AMGG decoction on lipolysis and non-shivering thermogenesis.

Brown and beige adipocytes have relatively higher mitochondrial ROS levels than white adipocytes, and ROS is essential in the non-shivering thermogenesis program of adipocytes [92]. However, its role is rather complicated since evidence points out that obesity accompanies pathologically increased ROS levels (or oxidative “stress”) and subsequent mitochondrial dysfunction in adipocytes [93,94,95]. Excessive ROS levels lead to the activation of NRF2, which regulates cellular responses to oxidative stress [96]. NRF2 is also essential for the increase of UCP1 expression in adipocytes upon β-adrenergic signaling [30]. HO-1 is also considered the first line of defense against oxidative stress. Increased expression of HO-1 enables cells to resist injury caused by ROS [97]. Furthermore, activation of the β3-adrenergic receptor induces not only UCP1 but also HO-1 expression in adipocytes [30]. HO-1 has been suggested to improve adipocyte function through the regulation of mitochondrial dynamics and biogenesis [98]. Here, we show that the antioxidant factors, NRF2 and HO-1, increase following the AMGB treatment (Figure 5D and Figure 6D). Considering the controversial action of ROS in adipocytes, these data suggest that the antioxidative effect of AMGB decoction is positive since the non-shivering thermogenesis is induced along with an elimination of excessive oxidative stress.

There were four different compositions of AMGB decoction used in this study. The specific proportion is usually the AMGB-1, which is composed of SP at 4 g, GF at 2 g, PC at 1 g, and EH at 3 g (total 10 g per preparation). The AMGB-2 is composed of SP at 4 g, GF at 2 g, PC at 3 g, and EH at 3 g (total 12 g per preparation); the AMGB-3 is composed of SP at 6 g, GF at 3 g, PC at 3 g, and EH at 6 g (total 18 g per preparation); and the AMGB-4 is composed of SP at 4 g, GF at 2 g, PC at 6 g, and EH at 6 g (total 18 g per preparation). Our results demonstrate that the AMGB-4 shows the highest potential in inhibiting adipogenesis and increasing lipolysis. There may be several reasons here. One of them could be the dose of preparation, since the AMGB-4 was prepared in the biggest dose along with the AMGB-3. In addition, a difference we noticed was the composition of EH. As described above, EH has been proven to have anti-obesity effects in animal and human data [56,57,58,59,60]. The AMGB-3 and AMGB-4 have the equal amount of EH in the formula. In fact, these two versions of AMGB decoction show similar effects. However, the AMGB-4 shows a better anti-adipogenic and lipolytic effect in iWAT (Figure 2C). This may be an effect from the PC content. Components of PC, such as evodiamine and berberine, are well known to suppress adipogenesis and induce lipolysis/thermogenesis [51,52,53,54].

**Figure 8:**
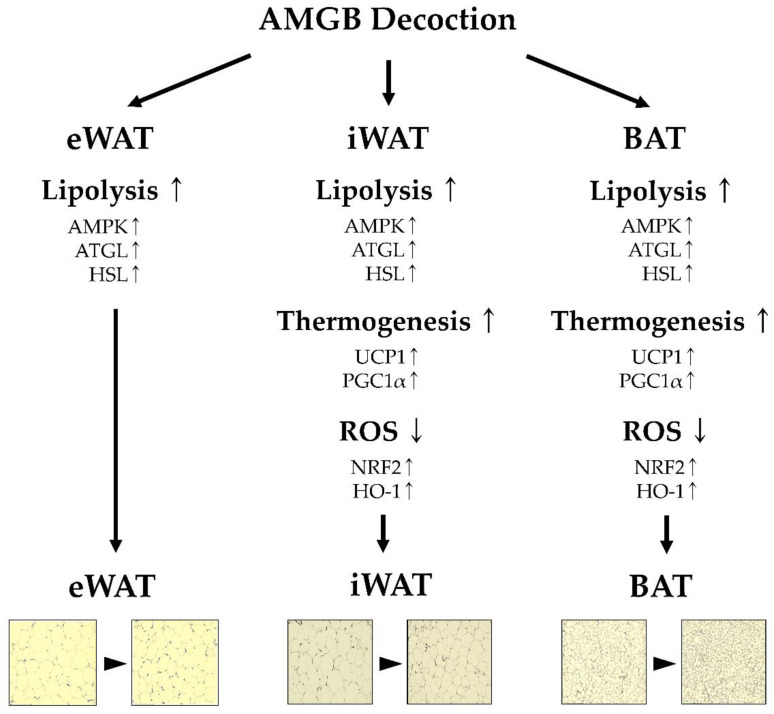
Schematic diagram of this study.

In conclusion, we herein demonstrate the anti-obesity effect of AMGB decoctions. In particular, the AMGB-4 decoction shows the most promising effects. Such effect is demonstrated in multiple pathways, including by suppressing adipogenesis in WAT, inducing lipolysis and non-shivering thermogenesis in BAT and iWAT, and showing protective antioxidant effects in response to excessive oxidative stress (Figure 8). This in vivo study supports the clinical use of AMGB decoctions to treat metabolic diseases.

## Figures and Tables

**Table 1:** Composition of the experimental diets (g/kg).

Formula	ND	HFD
Casein	200.0	265.0
L-Cystine	3.0	4.0
Corn Starch	397.486	-
Maltodextrin	132.0	160.0
Sucrose	100.0	90.0
Lard	-	310.0
Soybean Oil	70.0	30.0
Cellulose	50.0	65.5
Mineral Mix *^a^*	35.0	48.0
Calcium Phosphate	-	3.4
Vitamin Mix *^b^*	10.0	21.0
Choline Bitartrate	2.5	3.0
TBHQ, antioxidant *^c^*	0.014	-
Blue Food Color	-	0.1

*^a^* Mineral Mix, AIN-93G-MX (94046), containing (g/kg) calcium phosphate dibasic 500, sodium chloride 74, potassium citrate 220, potassium sulfate 52, magnesium oxide 24, manganous carbonate 3.5, ferric citrate 6, zinc carbonate 1.6, cupric carbonate 0.3, potassium iodate 0.0.1, sodium selenite 0.01, and chromium potassium sulfate 0.55. *^b^* Vitamin Mix, AIN-93-VX (94047), containing (g/kg) thiamin HCl 0.6, riboflavin 0.6, pyridoxine HCl 0.7, niacin 3, calcium pantothenate 1.6, folic acid 0.2, biotin 0.02, vitamin B12 (0.1 % trituration in mannitol) 1, dry vitamin A palmitate (500.00 U/g) 0.25, and manadione sodium bisulfite complex 0.15. *^c^* TBHQ: tertiary butylhydroquinone.

**Table 2:** Primary antibodies used in this study.

Primary Antibody	Manufacturer	Catalog No.	Molecular Weight
PPARγ	Cell Signaling Technology	2435s	53, 57
C/EBPα	Santa Cruz Biotechnology	sc-61	42, 30
ATGL	Abcam	EPR19650	55
p-HSL	Cell Signaling Technology	4139S	81, 83
HSL	Abcam	ab45422	90
p-AMPK	Cell Signaling Technology	2535s	62
AMPK	Cell Signaling Technology	2532s	62
UCP1	GeneTex	GTX112784	33
PGC1α	Thermo Fischer Scientific	PA5-38021	90
β-actin	Cell Signaling Technology	3700S	45
GAPDH	Santa Cruz Biotechnology	sc-32233	37

## Data Availability

The data presented in this study are available from the corresponding author upon request. The data are not publicly available due to currently ongoing patent application.
